# CRISPR-Cas9-Based Gene Knockout of Immune Checkpoints in Expanded NK Cells

**DOI:** 10.3390/ijms242216065

**Published:** 2023-11-08

**Authors:** Tahereh Mohammadian Gol, Miso Kim, Ralph Sinn, Guillermo Ureña-Bailén, Sarah Stegmeyer, Paul Gerhard Gratz, Fatemeh Zahedipour, Alicia Roig-Merino, Justin S. Antony, Markus Mezger

**Affiliations:** 1 Department of General Pediatrics, Hematology and Oncology, University Children’s Hospital Tübingen, 72076 Tübingen, Germany; tahereh.mohammadian-gol@med.uni-tuebingen.de (T.M.G.); hnah012@gmail.com (M.K.); paul.gratz@med.uni-tuebingen.de (P.G.G.); fatemeh.zahedipour@med.uni-tuebingen.de (F.Z.);; 2 MaxCyte Inc., Rockville, MD 20850, USA

**Keywords:** immune checkpoints, CRISPR-Cas9, natural killer cells, *A2AR*, *CBLB*, *NKG2A*, *CD96*

## Abstract

Natural killer (NK) cell immunotherapy has emerged as a novel treatment modality for various cancer types, including leukemia. The modulation of inhibitory signaling pathways in T cells and NK cells has been the subject of extensive investigation in both preclinical and clinical settings in recent years. Nonetheless, further research is imperative to optimize antileukemic activities, especially regarding NK-cell-based immunotherapies. The central scientific question of this study pertains to the potential for boosting cytotoxicity in expanded and activated NK cells through the inhibition of inhibitory receptors. To address this question, we employed the CRISPR-Cas9 system to target three distinct inhibitory signaling pathways in NK cells. Specifically, we examined the roles of A2AR within the metabolic purinergic signaling pathway, CBLB as an intracellular regulator in NK cells, and the surface receptors NKG2A and CD96 in enhancing the antileukemic efficacy of NK cells. Following the successful expansion of NK cells, they were transfected with Cas9+sgRNA RNP to knockout *A2AR*, *CBLB*, *NKG2A*, and *CD96*. The analysis of indel frequencies for all four targets revealed good knockout efficiencies in expanded NK cells, resulting in diminished protein expression as confirmed by flow cytometry and Western blot analysis. Our in vitro killing assays demonstrated that *NKG2A* and *CBLB* knockout led to only a marginal improvement in the cytotoxicity of NK cells against AML and B-ALL cells. Furthermore, the antileukemic activity of *CD96* knockout NK cells did not yield significant enhancements, and the blockade of A2AR did not result in significant improvement in killing efficiency. In conclusion, our findings suggest that CRISPR-Cas9-based knockout strategies for immune checkpoints might not be sufficient to efficiently boost the antileukemic functions of expanded (and activated) NK cells and, at the same time, point to the need for strong cellular activating signals, as this can be achieved, for example, via transgenic chimeric antigen receptor expression.

## 1. Introduction

NK cell therapies are considered an outstanding strategy in cancer immunotherapy, providing promising options to overcome the limitations of T-cell-based immunotherapy [1]. As innate immune system mediators, NK cells offer an “off-the-shelf” strategy for cancer treatment with high safety and no risk of graft-versus-host disease (GvHD) [2]. This is attributed to the unique ability of NK cells to target and eliminate cancer cells irrespective of MHC molecule presentation or the need for prior sensitization due to the presence of germline-encoded receptors [2]. NK cells mediate the killing process either through the direct release of lytic granules such as perforin and granzymes or by inducing apoptosis [3]. Moreover, NK cells secrete chemokines and cytokines such as interferon-gamma (IFN-γ), IL-10, CCL3, CCL4, and CCL5 to activate other immune cells [1,4]. A variety of inhibitory and activating surface receptors are present in NK cells. Following their binding to respective ligands, these receptors facilitate the transduction of activating or inhibitory signals, governing the regulatory mechanisms of NK cell functionality. The level of NK cell activity is determined by the balance or predomination of transduced activating or inhibitory signals [1,5]. In the clinic, using primary or expanded NK cells has advantages over NK cell lines. For instance, the poor expansion of NK-92 has been shown in vivo, which leads to transient effects and less efficiency in NK cell therapy [6].

Despite the numerous advantages of NK cells in cancer immunotherapy, there are certain limitations in their clinical application, including harvesting an adequate cell number, strategies employed by tumors to evade NK cell recognition, and difficulties associated with their infiltration into solid tumors [7,8]. It has been shown that the number of NK cells in cancer tissues is lower than in normal tissue, and they can be functionally suppressed in the tumor microenvironment [9]. Additionally, cancer cells can escape from NK cells by expressing inhibitory molecules and the immunosuppressive signals of NK cells [10]. An effective strategy to mitigate immunosuppression in cancer is the inhibition of pathways that negatively impact NK cell function. This approach has been extensively tested in preclinical and clinical trials in recent years, demonstrating encouraging results [11].

A2A adenosine receptor (A2AR), also known as ADORA2A, is a G-protein-coupled receptor (GPCR) with a high affinity for adenosine, expressed in several immune cells, including macrophages, NK cells, and T cells, and mediates the generation of adenosine from ATP. It has been shown that A2AR-mediated adenosine signaling blocks the antitumor activity of T and NK cells [12]. E3 ubiquitin ligase Cbl-b (Casitas B cell lymphoma-b) is known to be a negative regulator of immune activation. This protein plays a critical role in inhibitory signaling for the regulation of protein tyrosine kinases (PTKs), T and B-cell immune tolerance, and T-cell activation. Nonetheless, little is known about the exact role of CBLB in NK cell function [13,14,15,16]. Its regulatory function seems to be associated with the TAM receptor family, blocking NK cell activation via the ubiquitylation of LAT1 [17,18].

CD96 (TACTILE) is a member of the immunoglobulin superfamily and interacts with nectin and nectin-like proteins [19]. A high level of CD96 has been reported in myelodysplastic syndrome, acute myeloid leukemia (AML), and T-cell acute lymphoblastic leukemia (T-ALL) [20,21]. It is also defined as a cell surface marker for AML stem cells [19]. CD94/NK group 2 member A (NKG2A) is a tyrosine-based inhibitory motif (ITIM) and is expressed at the cell surface of both T and NK cells as a heterodimer with CD94 in humans [22]. NKG2A is the most prominent NK inhibitory receptor, bindinghuman leukocyte antigen (HLA)-E and blocks T and NK cell cytotoxicity [22,23]. In a previously published study, we showed that HLA-E is highly expressed in acute myeloid leukemia (AML) and B-cell acute lymphocytic leukemia (B-ALL) cell lines [24]. AML and ALL are two subsets of acute leukemia with high frequency, subsequently, in adults and children [25,26].

Taking these factors into account, we developed a CRISPR-based gene knockout method to target *A2AR, CBLB, CD96*, and *KLRC1 (NKG2A)* genes to evaluate their antitherapeutic role in AML and B-ALL cancer.

## 2. Results

### 2.1. Primary NK Cell Isolation, Expansion, and Receptor Profile Characterization

**Figure 1:**
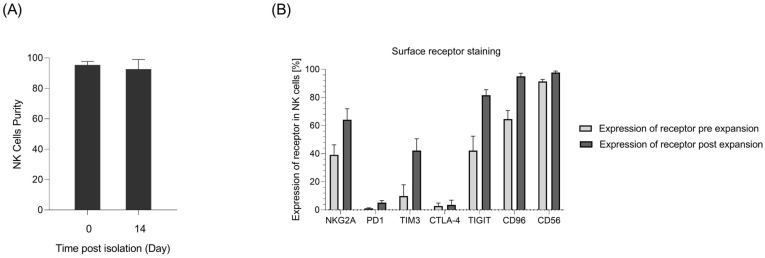
Primary NK cell isolation, expansion, and receptor profile characterization. (**A**) NK cell purity on the day of isolation and 14 days post-expansion from merged donors measured as CD56+ cells in flow cytometry analyses (*n* = 6). (**B**) Flow cytometry analysis of receptor expression in NK cells pre- and post-in vitro expansion (*n* = 8).

NK cells constitute 5–20% of all circulating lymphocytes in humans and can be identified by the presence of CD56 and the absence of CD3 on their surfaces [27]. Despite this small fraction, NK cells need to be infused in large numbers in patients. To acquire this huge number, an optimal protocol has to be applied for the expansion of NK cells ex vivo [28]. In this study, immunomagnetic negative selection was used to target non-NK cells and isolate NK cells. Using this method, more than 95% NK cell purity was achieved after isolation (Figure 1A). For the expansion of NK cells, isolated cells were incubated with an irradiated K562mb15-41BBL cell line as feeding cells. K562mb15-41BBL is a myeloid leukemia cell line that is genetically modified to express membrane-bound interleukin-15 and 41BB ligands in order to specifically activate NK cells but not T lymphocytes. Flow cytometry analysis showed more than 90% purity for NK cells 14 days after incubation. The proliferating rate of NK cells was monitored within 14 days via counting, and the expansion rate was calculated over 100 times on day 14 (Figure 1B).

To assess the expression level of different receptors in NK cells, receptor profile characterization was performed using flow cytometry. The data showed an increase in the expression of the investigated inhibitory receptors, namely, NKG2A, TIM3, TIGIT, and CD96, following the expansion of NK cells. In the meantime, PD-1 expression was maintained by NK cells at a low level (Figure 1).

### 2.2. Targeting Inhibitory Signals Involved in NK Cell Function Using CRISPR-Cas9 System

The MaxCyte GTx^®^ instrument (MaxCyte Inc, Rockville, MD, USA) is GMP-compliant and clinically validated, enabling effortless translation into the clinic. Here, we used the MaxCyte GTx^®^ to deliver CRISPR-Cas to difficult-to-transfect expanded NK cells. Initially, to optimize the electroporation, DsRed mRNA was transfected into expanded NK cells and the NK-92 cell line (control) using different programs. The data showed the efficient expression of DsRed using programs 4 and 5 in both expanded NK cells (up to 96%) and NK-92 cells (up to 99%) (Supplementary Figure S1).

**Figure 2:**
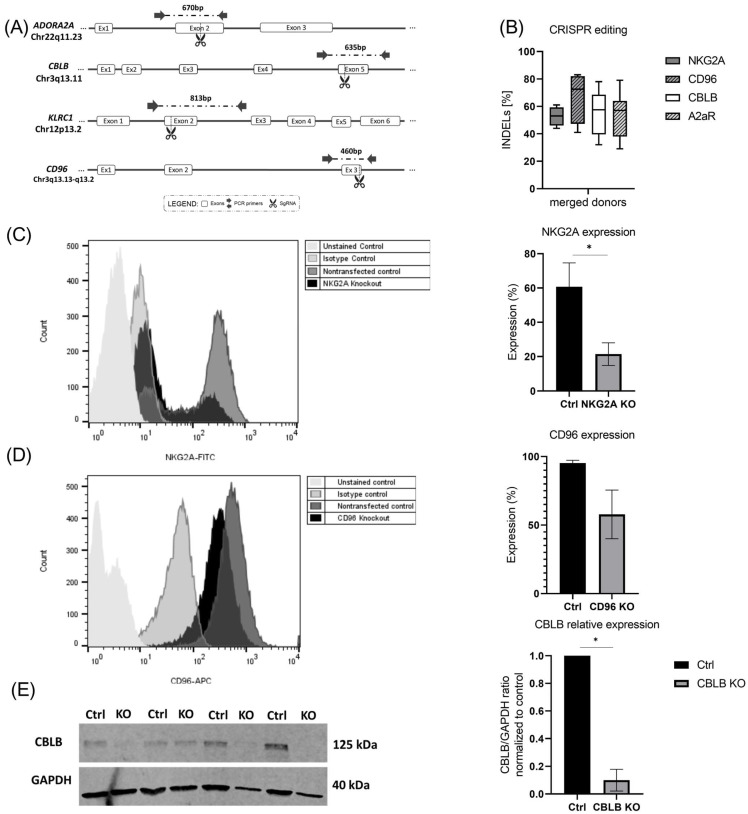
CRISPR-Cas9-based knockout in expanded NK. (**A**) Schematic illustration showing the cutting sites of Cas9 in *A2AR* and *CD96* genes, as well as *NKG2A* and *CBLB* (adapted from previous publication) [24]. (**B**) Expanded NK cells were transfected with RNP complexes containing sgRNA targeting *NKG2A, CD96, CBLB*, and *A2AR*. After Sanger sequencing, indel frequencies were analyzed using the ICE v3 software (www.ice.synthego.com, (accessed on July 2022 to July 2023)). (**C**,**D**) NKG2A and CD96 receptor expression in NK cells after knockout was measured using flow cytometry. (**E**) Immunoblot of CBLB and GADPH in non-transfected NK cells and transfected cells. (**E**) Normalized CBLB/GADPH ratio of band densitometry in non-transfected and transfected NK cells. * *p* < 0.05.

To check the effect of CRISPR-Cas9-based gene knockout on the inhibitory signaling pathways of expanded NK cells, different sgRNAs were applied to target *A2AR*, *CBLB*, *NKG2A*, and *CD96* genes in expanded NK cells. The selected sgRNAs were either previously published [14,29,30] or newly designed by our group using CHOPCHOP, considering high knockout efficiency and low off-target ranking. Figure 2A represents the target region of each sgRNA in the related gene sequence. Then, 14–15 days after expansion, cells were electroporated with Cas9-sgRNA RNP, and 3 days later, Sanger sequencing was performed. The results of Sanger sequencing showed up to 61% indel frequency for *NKG2A*, 83% for *CD96*, 69% for *CBLB*, and for 79% *A2AR*. The mean value of the indels for merged donors is shown in Figure 2B. Five days after electroporation, cells were harvested to check for knockout efficiency at the protein level using flow cytometry or Western blot (Figure 2C–E). Given the nonspecific binding of the antibody to Fc receptors, staining for A2AR in NK cells was not successful, although using the same sgRNA and antibody in NK-92 cells showed a reduction in the A2AR protein after knockout with 80% indel frequency (Supplementary Figure S2).

### 2.3. CRISPR-Mediated Blocking of CD96 and NKG2A, Two Inhibitory Receptors in NK Cells

In our previous publication, we showed that *NKG2A* knockout did not increase the cytotoxicity of the NK-92 cell line either in parental cells or in CD19-CAR- and CD276-CAR-induced NK-92 cells [24]. Here, we evaluated the effect of the CRISPR knockout of *NKG2A* on expanded NK cells by performing a cytotoxicity luciferase assay using U937 CD19 tag/Luc and Nalm-6 GFP/Luc cells. U937 is a pro-monocytic human myeloid leukemia cell line [31], transduced to express luciferase and Nalm-6Luc/GFP cells generated from the human precursor B-cell lymphoblast cell with the stable introduction of a GFP-firefly luciferase expression construct.

**Figure 3:**
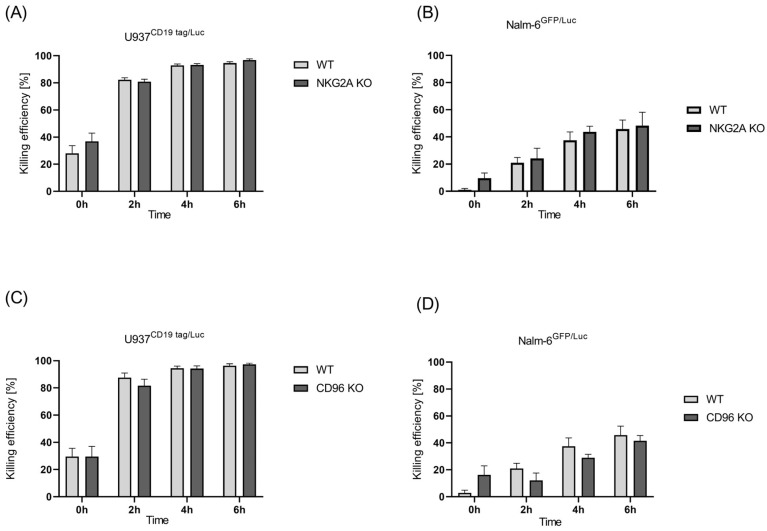
Killing efficiency of unmodified primary NK cells vs. edited *NKG2A* and *CD96* cells. Luciferase assay was performed to check for cytotoxicity of *NKG2A* KO NK cells against (**A**) U937 CD19 tag/Luc and (**B**) Nalm-6 GFP/Luc and *CD96* KO cells against (**C**) U937 CD19 tag/Luc and (**D**) Nalm-6 GFP/Luc for a time span of 6 h at a 5:1 E:T ratio. Specific lysis is shown as mean ± SE (*n* = 3).

The results of the killing assay showed that a modest and statistically insignificant improvement was observed in *NKG2A* knockout NK cells compared with wild-type cells against the B-ALL and AML cells (Figure 3A,B). Next, the generated *CD96* knockout NK cells were incubated with AML and B-ALL cells. Again, *CD96* knockout did not improve the killing function of the NK cells. Yet, in some donors, even a reduction in killing was observed against both U937 CD19tag/Luc and Nalm-6 GFP/Luc cells (Figure 3C,D).

### 2.4. CRISPR-Mediated Blocking of CBLB as Intracellular Regulator of NK Cells

**Figure 4:**
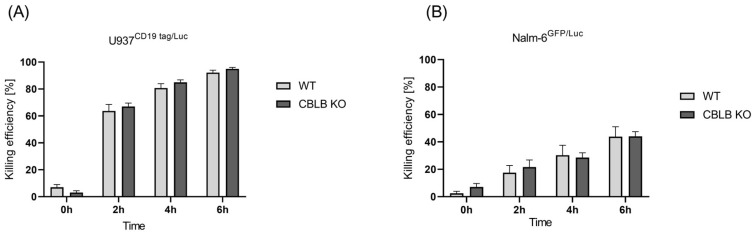
Killing efficiency of unmodified primary NK cells vs. edited CBLB cells. Luciferase assay was performed to check for cytotoxicity of *CBLB* KO NK cells against (**A**) U937 CD19 tag/Luc (*n* = 4) and (**B**) Nalm-6 GFP/Luc (*n* = 3) at 0, 2, 4, and 6 h after coculture at a 5:1 E:T ratio. Specific lysis is shown as mean ± SE.

After the validation of *CBLB* knockout at the genetic and protein levels in NK cells, we aimed to check the cytotoxicity of edited NK cells against AML cells (U937 CD19tag/Luc) in vitro and compared this with wild-type cells. The cytotoxic performance of *CBLB*-KO cells against the AML cell line showed a slight but insignificant increase at 2 h, 4 h, and 6 h post-incubation compared with wild-type NK cells (Figure 4A). In contrast, *CBLB* knockout could not boost the antileukemic activity of NK cells against B-ALL cells (Figure 4B). These data demonstrate that although the CRISPR-based blocking of *CBLB* could improve the killing performance of primary T cells against Nalm-6 GFP/Luc, and especially U937 CD19tag/Luc cells (Supplementary Figure S3), it showed only a slight improvement in the killing efficacy of NK cells against these cell lines in vitro.

### 2.5. The Effect of Blocking Metabolic Purinergic Signaling on Antileukemic Function of NK Cells

**Figure 5:**
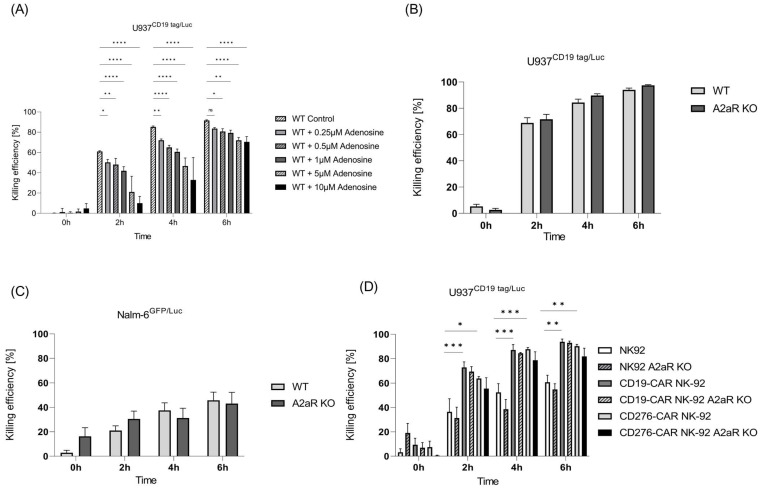
Role of adenosine and its receptor A2AR in killing performance of NK cells. (**A**) Killing performance of NK cells in the presence of extracellular adenosine was evaluated in vitro via luciferase assay at different time points (*n* = 3). Killing efficiency of unmodified primary NK cells vs. edited *A2AR* cells against (**B**) U937 CD19 tag/Luc (*n* = 6) and (**C**) Nalm-6 GFP/Luc (*n* = 3) at 0, 2, 4, and 6 h after coculture at a 5:1 E:T ratio. (**D**) Luciferase killing assay for parental and *A2AR* KO NK-92, CD19-CAR-NK-92, and CD276-CAR-NK-92 cell lines against U937 CD19 tag/Luc cells (*n* = 3). Specific lysis is shown as mean ± SE. * *p* < 0.05; ** *p* < 0.01; *** *p* > 0.001; **** *p* < 0.0001, ns, non-significant (*p* > 0.05).

To evaluate the cytolytic capabilities of expanded NK cells in the presence of adenosine, a cytotoxicity luciferase assay was performed. To this end, NK cells were treated with different concentrations of adenosine in the assay medium and subsequently incubated with U937 CD19tag/Luc cells. As shown in Figure 5A, a significant adenosine con-centration-dependent reduction was observed for the killing performance of NK cells against U937 CD19tag/Luc. This effect was mostly discernible at 2 and 4 h post-coculture of the effector and target cells. In contrast, the cytotoxicity of NK cells against the B-ALL cell line (Nalm-6GFP/Luc) was not affected by extracellular adenosine amount, and no improvement was observed in *A2AR*-depleted NK cells (Figure 5B,C).

We earlier showed that the presence of chimeric antigen receptors such as CD19-CAR and CD276-CAR can strongly improve the cytotoxic effect of NK-92 cells [24]. To check if *A2AR* knockout can induce further killing improvement in CAR-induced NK-92 cells, a killing assay was performed. As expected, CD19- and CD276-CAR-induced NK-92 cells exhibited better killing performance than parental cells. However, *A2AR* knockout could not further increase their killing capacity (Figure 5D).

## 3. Discussion

Immunotherapy has recently emerged as the preferred strategy for cancer treatment owing to its potential to elicit long-lasting responses, mild levels of side effects, and the promising prospect of enhancing patient survival and overall quality of life [11,32,33]. NK-cell-based immunotherapy is widely considered a safe and effective therapy for solid tumors and hematologic malignancies [34]. In cancer clinical trials, a massive number of NK cells are required (ranging from 5 × 10^6^ to 5 × 10^7^ CD3^−^CD56^+^ NK cells per kilogram) [28]. Given the low number of NK cells in peripheral blood, an optimal ex vivo expansion of NK cells is required. It has been shown that ex vivo-expanded NK cells exhibit higher cytokine secretion profiles and have stronger cytotoxicity against tumor cells, and the maintenance of this activation depends on the presence of IL-15 or IL-2 [35]. Moreover, Mao et al. demonstrated a critical role for IL-15 in the antileukemic function of NK cells, supporting the incorporation of IL-15 in the expansion phase or through low-dose infusions following NK cell transfer in clinical settings [36]. In our study, utilizing K-562mb-IL-15 cells as feeder cells, we achieved a high expansion ratio for NK cells (up to 173.6×). Additionally, through the activation of these cells using IL-15 and IL-2, we promoted their effector function. These dual advantages overcome the limitation of using primary NK cells in terms of insufficient quantity and poor post-infusion persistence in the clinic.

Leukemia is defined as a collection of hematologic malignancies marked by the sustained proliferation of leukemic cells originating from hematopoietic stem cells affecting blood and bone marrow [11]. Enhancing the expression of inhibitory ligands and receptors is one of the mechanisms by which leukemic cells can escape from NK cell response [37,38]. Therefore, one approach to reverse NK cell dysfunction in tumors is blocking inhibitory pathways to increase the antitumor immunity of NK cells. Several monoclonal antibodies are currently employed to block NK cell checkpoint inhibitors in preclinical studies and clinical trials for leukemia [11].

CD96 and NKG2A are known as inhibitory receptors of NK and T cells. In this study, the killing capacity of expanded NK cells after the CRISPR-mediated knockout of these two receptors was addressed. We previously showed that *NKG2A* knockout failed to improve the killing performance of the NK-92 cell line against melanoma, AML, and B-ALL cells [24,29]. Bexte et al. performed CRISPR-based knockout in primary NK cells and reported the enhancement of tumor cell lyses in *NKG2A*-KO cells in comparison with WT NK cells against multiple myeloma cells after 24 h of coculturing in the FACS-based cytotoxic assay [39]. In contrast to Bexte et al. but similar to our previous publications [24,29], in this study, no significant improvement was observed in the killing of AML and B-ALL cells by *NKG2A*-KO-expanded NK cells, showing that NK cells might have different levels of cytotoxicity against different cancer cells.

Furthermore, *CD96* knockout not only showed no increase in the cytotoxicity of NK cells against AML cells but also resulted in an insignificant reduction in killing against B-ALL cells. This observation is inconsistent with Stanietsky et al., where anti-CD96 mAb-treated NK cells could not redirect NK cytotoxicity independently and showed a slight reduction in specific lysis [40]. Other studies have reported the negative role of CD96 in the antitumor function of NK cells in mouse models [41,42], although it has been revealed that this effect is due to increased IFNγ production in *CD96* knockout mice but not because of the enhancement of NK-cell-mediated cytotoxicity. Chan et al. showed the enhancement of cytokine production using NK cells in Cd96−/− mice in vivo; however, they did not observe any difference between mice with Cd96−/− and wild-type NK cells in terms of cytotoxicity in vitro, suggesting that CD96 signaling might not be strong enough to block cytotoxicity [41]. Moreover, it has been shown that cytotoxic lymphocytes have different activation thresholds for cytotoxicity and IFN-γ production [43]. This can explain the conflicting in vitro data from our group and others.

CBLB is an E3 ubiquitin ligase, and its upregulation and negative role in activated human NK cells have been reported [16]. Lu et al. showed that the siRNA-mediated downregulation of *CBLB* of primary NK cells resulted in an increase in cytotoxic activity against leukemia cell lines, including MV4-11 and Molm-13, in a ^51^Cr cytotoxicity assay [16]. However, in their study, one of the AML cell lines did not show this effect in the absence of IL-15, emphasizing the relevance of culture conditions and the type of target cells for the level of cytotoxicity evinced by NK cells. Guo et al. used the CRISPR-Cas9 system to deplete *CBLB* in placenta-derived CD34+ hematopoietic stem cells and differentiated them into PNK cells. They showed that *CBLB* knockout PNK cells have higher cytotoxicity against the myeloma cell lines RPMI8226 and U266 and the plasma cell leukemia cell line ARH77 [30]. In our study, CRISPR-Cas9-based *CBLB* knockout was applied to expanded NK cells from peripheral blood and showed only a slight improvement in cytotoxicity against U937 cells. Likewise, a previous study by our group showed that *CBLB* knockout alone is not enough to boost antileukemic activity in NK-92 cells against the B-ALL cell line [24]. However, *CBLB* knockout in T cells showed a significant improvement in killing performance against the U937 and Nalm-6 cell lines (Supplementary Figure S3), proving the efficiency of the applied CRISPR-Cas9 system and killing assay in our study.

The purine nucleoside adenosine is an immunosuppressive metabolite, physiologically present at very low concentrations in body fluids, and is crucial for regulating excessive immune responses during inflammation and tissue damage [44]. A2AR, expressed in leukemic and immune cells, is the main receptor that transfers signals from extracellular adenosine to apply autocrine and paracrine effects [45]. We demonstrated the significant effect of extracellular adenosine on the killing performance of expanded NK cells against AML cells (Figure 5A), indicating the role of adenosine in suppressing immune function against AML cells. It has been shown that targeting the A2AR signaling pathway by blocking A2AR alone or in combination with other molecules can restore immune competence in vivo in leukemia mouse models [45,46]. Brauneck et al. previously reported an increase in the NK-cell-mediated lysis of AML cells (MV-4-11, TF-1, and OCI-AML3) by blocking A2AR in the NK-92 cell line in vitro, which was more notable after the combinational blockage of A2AR and TIGIT [47]. These data contrast with our results, as no improvement was observed in killing efficiency after the knockout of *A2AR* in NK-92 cells. However, Brauneck et al. used a different method (A2AR antagonism) for blocking A2AR and different AML cell lines as target cells. Moreover, our results showed that the CRISPR-based blocking of A2AR has a minor and insignificant effect on the NK-cell-mediated killing of AML cells.

In our experiment, Fc receptors, staining for A2AR in NK cells, were not successful, although a reduction in A2AR expression in edited NK-92 cells after CRISPR-based knockout proves that the applied sgRNA can successfully target the *A2AR* gene. It is worth noting that difficulties in staining A2AR in primary cells were reported by another study authored by Giuffrida et al., who stated in their publication that they could not show the *A2AR* knockout efficiency of T cells because of a lack of A2AR antibodies suitable for flow cytometry [48]. This discrepancy could be attributed to the antibody’s specificity to particular subpopulations of NK cells, which might be predominant in NK-92 cells but less in primary NK cells. Moreover, differences in post-translational modifications of the target protein in the two cell types can affect antibody binding. Finally, NK-92 cells may express the target receptor at higher levels or in a different conformation compared with primary NK cells, making it more accessible to the antibody [49,50].

Taken together, previous data show that mixed results have been observed for the mentioned targets in improving NK cell tumor-killing efficacy. In the current study, there are several key differences compared with the published data in terms of diverse effector cells; various target cells; different cytotoxicity assays; differences in media, cytokines, and culture conditions; and cell manipulation. The central scientific question of this study is whether blocking inhibitory receptors/pathways can further increase and preserve the cytotoxicity of expanded (and activated) NK cells. To the best of our knowledge, this is the first work that has analyzed the effect of the CRISPR-mediated knockout of *NKG2A, CD96, CBLB,* and *A2AR* in expanded NK cells against the U937 CD19/Luc (AML) and Nalm-6 GFP/Luc (B-ALL) cell lines. One of the central factors might be the expansion of NK cells using K562-mbIL15 cells as feeder cells for 14 days, through which they become strongly activated in advance of editing. We speculate that this activation cannot be further boosted by CRISPR-mediated knockouts of *NKG2A, CD96, CBLB*, and *A2AR*. A similar observation was reported for another target (Tim3) by Audenaerde et al., where the authors stimulated NK cells with IL-15 and showed elevated NK-cell-mediated killing against pancreatic stellate cells (PSCs), although blocking Tim3 in the activated cells could not change the killing capacity [51]. This idea is supported by the observation that, in primary T cells, we could confirm previous publication data [52] with enhanced killing activity after *CBLB* knockout (Supplementary Figure S3). The data show that our methods and model work in assessing the enhancement of the tumor-killing capability of primary cells.

Overall, in the present study, we could successfully expand primary NK cells with high purity and efficiency. This study underscores the potential of the CRISPR-Cas9 system to edit expanded NK cells. However, further investigation is needed to address the exact therapeutic effects of targeting *A2AR, CBLB, NKG2A*, and *CD96* in NK cells. Each of these proteins is part of a complex network that warrants comprehensive exploration in studies involving diverse cancer cell types. Moreover, sorting out the low-expression cells after CRISPR knockout and performing cytotoxicity tests using 100% knockout cells may lead to more convincing conclusions. Consequently, to attain a better understanding of the therapeutic potential of each target, it is advisable to conduct analyses using NK cells derived from patients with the specific cancer type.

## 4. Materials and Methods

### 4.1. Cell Line Cultures

K-562mb-IL-15, U937CD19/Luc, and Nalm-6 GFP/Luc tag were cultured in RPMI 1640 medium supplemented with 10% heat-inactivated fetal bovine serum (FBS) and 1% penicillin/streptomycin at 37 °C in a humidified 5% CO_2_ incubator.

### 4.2. Primary Human NK Cell Isolation and Culture

Heparinized peripheral blood samples were obtained from healthy donors after informed consent. Peripheral blood mononuclear cells (PBMCs) were subsequently isolated using Ficoll-density gradient centrifugation (BioColl^®^ Separation Solution, Bio&SELL GmbH, Nuremberg, Germany) according to the manufacturer’s instructions. Isolated PBMCs were washed twice with PBS via centrifugation before evaluating the NK cell frequency with flow cytometry. CD56+ NK cells were isolated via negative selection using the EasySep™ Human NK Cell Isolation Kit (StemCell Technologies, Saint Égrève, France) according to the manufacturer’s instructions.

Isolated NK cells were cultured with irradiated K562-mb15 cells as feeder cells at a 0.66:1 ratio and maintained in NK cell medium (RPMI-1640 medium supplemented with 1% penicillin/streptomycin, 1% L-glutamine, 10% human serum, and 100 IU/mL of human recombinant IL-2 (Miltenyi Biotec, Bergisch Glattbach, Germany)) for 14 days at 37 °C and 5% CO_2_. In total, 5 ng/mL of soluble IL-15 was added to the medium after day 14.

### 4.3. Flow Cytometry

Percentages of the whole blood (WB) cell compartment containing NK cells were determined after PBMC isolation and isolation of NK cells on day 0. For the receptor analysis, harvested cells were washed and resuspended in serum-free and azide-free PBS and were characterized with specific A2AR-PE (Bio-Techne GmbH, Wiesbaden-Nordenstadt, Germany), CD3-PE, CD56-APC, CD96-APC, CTLA-4-PE, NKG2A-FITC, PD1-PE, TIGIT-PE, or TIM3-APC (Miltenyi Biotec, Bergisch Gladbach, Germany) fluorophore-labeled antibodies for 30 min at room temperature in the dark. An isotype control was used for each fluorophore used. Samples were run on a BD FACSCalibur™ flow cytometer (BD Biosciences, San Diego, CA, USA) and analyzed with the FlowJo-10 software (FlowJo LLC., BD Biosciences, Franklin Lakes, NJ, USA).

### 4.4. CRISPR-Cas9 Transfection

The sgRNAs were selected either from previous publications or were newly designed using the CHOPCHOP software, version 3 (www.chopchop.cbu.uib.no (accessed on June 2022); see Table 1. Before CRISPR/Cas9 electroporation, the expanded NK cells were harvested, centrifuged at 400 G for 5 min, and washed with PBS. To create the RNP complex, 1.5 µL of sgRNA and 1.23 µL of Cas9 were mixed and incubated for 15 min at room temperature. In total, 2.5 × 10^6^ NK cells were resuspended in 50 µL of MaxCyte electroporation buffer and then mixed with RNP solution. The NK cells were electroporated with the MaxCyte GTx^®^ instrument using the NK-4 and -5 protocols. Following electroporation, the cells were transferred to a pre-warmed 6-well plate. After 30 min of recovery time, 1.25 mL of warmed RPMI 1640 medium with 10% human serum, 1 mM of glutamine without antibiotics, and interleukins were added to the cells. Following a recovery period of 4 h, each well received 1.25 ml of RPMI medium with 200 U/mL of penicillin, 200 mg/L of streptomycin, IL-2 (200 U/mL), and IL-15 (100 U/mL). As a transfection control, DsRed mRNA was electroporated to the cells in all experiments, as described before [53].

### 4.5. Evaluation of CRISPR-Cas9-Induced Knockout

Three days after transfection, 5 × 10^5^ transfected cells and WT NK cells were harvested to isolate DNA using the NucleoSpin^®^ DNA purification Kit (Macherey Nagel, Dueren, Germany). The target sequence containing *CBLB, CD96, NKG2A*, or *A2aR* was amplified through PCR, with their respective primer sequences shown in Supplementary Table S1. Then, all the samples were submitted for Sanger sequencing, and the resulting data were analyzed using Synthego’s ICE v3 software to confirm the gene editing (https://ice.synthego.com (accessed on July 2022 to July 2023)).

### 4.6. Western Blot

Approximately 3 × 10^6^ cells were suspended in RIPA Lysis (with 1× Protease and Phosphatase Inhibitor Cocktail (ThermoFisher, Waltham, MA, USA)). All samples were incubated on ice for 20 min. The soluble fraction was then obtained via centrifugation at 10,000× *g* for 10 min at 4 °C. The standard Bradford assay was used to measure protein concentration. In total, 20 µg of protein was loaded onto a Mini-PROTEAN TGX gel (Bio-Rad, Fort Worth, TX, USA), and the gel was blotted onto a Midi format 0.2 µm PVDF membrane utilizing the Trans-Blot Turbo Transfer System (Bio-Rad). The membrane was blocked with EveryBlot Blocking Buffer (Bio-Rad) and incubated for 1 h at room temperature (RT) or overnight at 4 °C with primary antibodies (rabbit anti-CBLB diluted at 1:250 and rat anti-GAPDH diluted at 1:1000 in blocking buffer). Following washing with PBS-T, the membrane was incubated for 1 h at room temperature with the secondary antibodies (IRDye 800CW goat anti-rat and IRDye 680RD goat anti-rabbit at 1:15,000 in a blocking buffer). The membrane was developed utilizing LI-COR.

### 4.7. Luciferase-Based Cytotoxicity Assay

The cytotoxicity of effector cells (WT and KO NK cells) against target cells (U937 CD19/Luc or Nalm-6Luc/GFP) was evaluated using a luciferase-based cytotoxicity assay. U937 CD19/Luc and Nalm-6Luc/GFP are capable of metabolizing luciferin and expressing luciferase, which was added at specific time intervals during the assay. In total, 1 × 10^4^ target cells (U937 CD19/Luc or Nalm-6Luc/GFP) were resuspended in 100 µL of assay medium (RPMI 10% FBS10% FBS, 1% L-glutamine, and 1% penicillin/streptomycin), and 5 × 10^4^ effector cells (WT or KO NK cells) were resuspended in 50 µL of assay medium and cocultured on a black 96-well plate. At 0, 2, 4, and 6 h, D-luciferin (Gold-Bio) was added to each well for measurement. For the preparation of the working assay solution for D-luciferin, a stock aliquot was mixed 1:4 in assay medium, and 50 µL was added to each assay well. The samples were directly evaluated for their cytotoxicity with the luminescence measurement in a TECAN Spark reader after adding D-luciferin.

To calculate the specific lysis by effector cells, the following formula was used:

$$\%\;\mathrm{specific}\;\mathrm{lysis}=\frac{\mathrm{measured}\;\mathrm{value}\;-\;\mathrm{spontaneous}\;\mathrm{lysis}\;\mathrm{mean}}{\mathrm{maximum}\;\mathrm{killing}\;-\;\mathrm{spontaneous}\;\mathrm{lysis}\;\mathrm{mean}}\times 100$$

### 4.8. Statistical Analysis

All statistical analyses were conducted using Prism 8.0 (GraphPad Software, La Jolla, CA, USA) on at least biological and experimental triplicate datasets. Two-way ANOVA analyses and non-parametric t-tests were performed for, respectively, Gaussian-distributed datasets and the normally distributed data (analyzed with the Shapiro–Wilk test). The FlowJo-10 software (FlowJo LLC, Ashland, OR, USA) was used for the analysis of flow cytometry data.

## Figures and Tables

**Table 1:** List of used sgRNA sequences for target disruption.

Name	Sequence (5′-3′)	Source
*A2AR*	CTCCACCGTGATGTACACCGAGG	Jones et al. [54]
*CD96*	GTTCACCTATGCATGGTCGGTGG	Own design (CHOPCHOP)
*CBLB*	TAATCTGGTGGACCTCATGA	Guo et al. [30]
*NKG2A*	GGTCTGAGTAGATTACTCCT	Grote et al. [29]

## Data Availability

The data presented in this study are available from the corresponding authors upon reasonable request.

## Supplementary Materials

The following supporting information can be downloaded at https://www.mdpi.com/article/10.3390/ijms242216065/s1.
