# Supplementary material for: CRISPR-Cas9-Based Gene Knockout of Immune Checkpoints in Expanded NK Cells
Source: Int J Mol Sci. 2023 Nov 8;24(22):16065. doi: 10.3390/ijms242216065 (PMC10671270; doi:10.3390/ijms242216065)
Supplement: Supplementary file 1 [file ijms-24-16065-s001.zip › ijms-2665097-supplementary.pdf]

Supplementary data.

Table S1: List of primers for DNA amplification.

Name	Sequence (5′-3′)	Annealing temperature
A2aR*-for**<br>A2aR*-rev**	GCCAAGTGTGGGGTAAGGG<br>AATGTAGCGGTCAATGGCGA	59°C
CBLB*- for**<br>CBLB*-rev**	TGATAGCCTAGGACTGTTTGAGAGAA<br>GTTATCAGATGCTGTGAGCCTGG	55°C
CD96*-for**<br>CD96*-rev**	TGAGGGCTATCGTGGGAGTT<br>GGCTTGCCTGTGAGAAGGAA	59°C
NKG2A*-for**<br>NKG2A*-rev**	TACTCGTTCTCCACCTCACC<br>TAACGTGAAAATTCCCCTTGTAATC	55°C

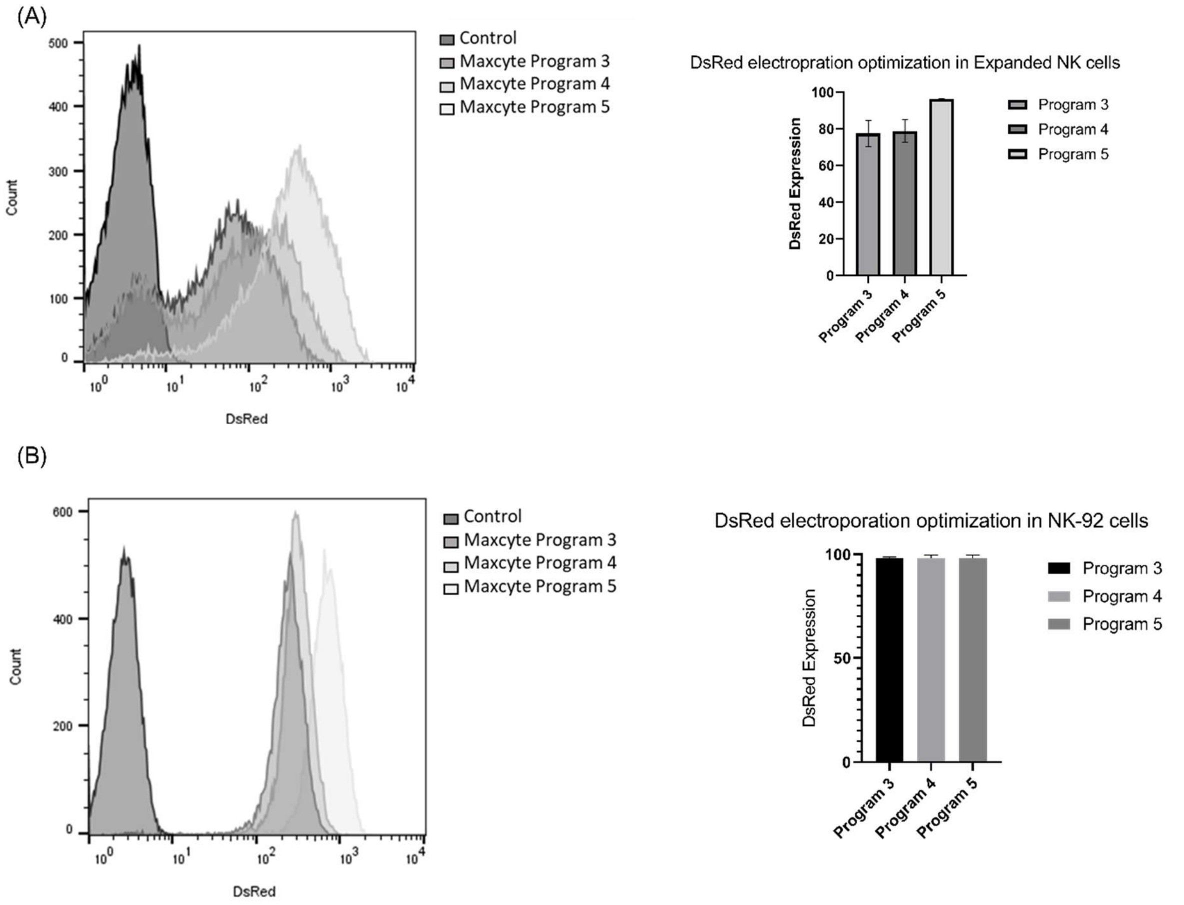

Figure S1. Optimization of DsRed mRNA electroporation. A) Expanded NK cells and B) NK-92were electroporated with DsRed mRNA using three different programs for NK cells on the MaxCyte GTx® electroporator. Expression of DsRed was measured by flow cytometry (n=2).

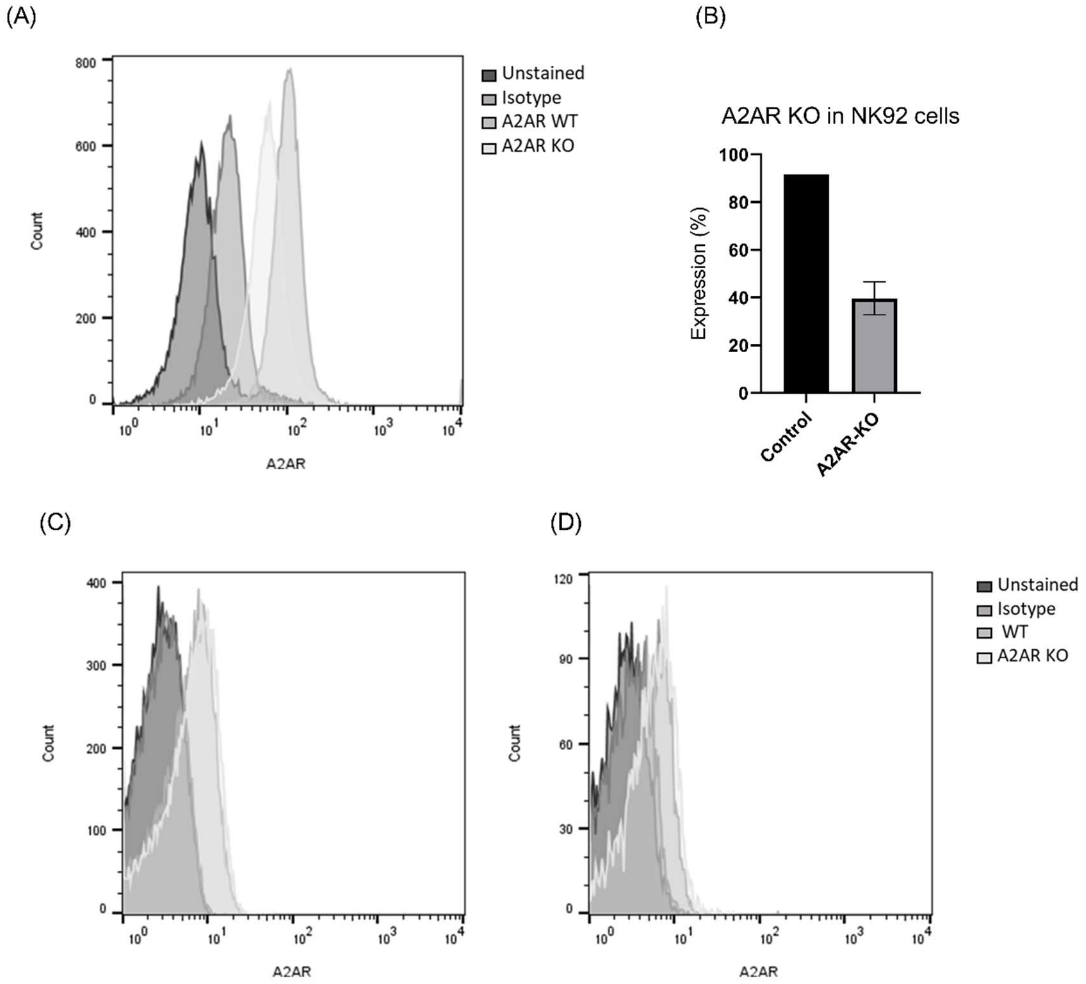

Figure S2. A2AR expression on NK92 cells. A and B) A2AR expression on NK92 cells C) on expanded NK cells before FC blocking and D) after FC blocking on expanded NK cells was measured using flow cytometry after A2AR knockout.

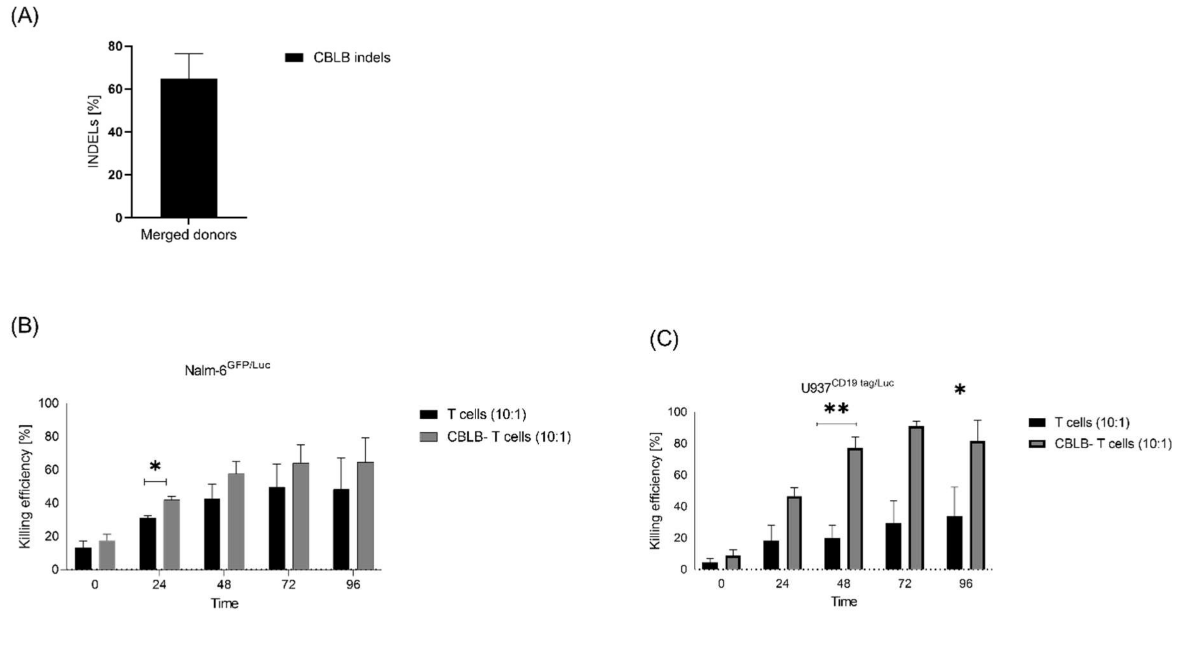

Figure S3. CBLB knockout in T cells. A) indel frequency after CBLB KO in merged donors (n=5). Luciferase assay was performed to check for cytotoxicity of CBLB KO T cells against B) U93719 tag/Luc (n = 4) and C) Nalm-6 GFP/Luc (n = 3) at 0,22,48,72 and 96 h after coculture at 10:1 E:T ratio. Specific lysis is shown as mean ± SE. * $p < 0.05$; ** $p < 0.01$.
